# Supplementary material for: Phylogenetically and functionally diverse microorganisms reside under the Ross Ice Shelf
Source: Nat Commun. 2022 Jan 10;13:117. doi: 10.1038/s41467-021-27769-5 (PMC8748734; doi:10.1038/s41467-021-27769-5)
Supplement: Supplementary file 1 — Supplementary Information file (PDF) [file 41467_2021_27769_MOESM1_ESM.pdf]

Supplementary information for

Phylogenetically and functionally diverse microorganisms reside under the Ross Ice Shelf

Authors: Clara Martínez-Pérez, Chris Greening, Sean K. Bay, Rachael J. Lappan, Zihao Zhao, Daniele De Corte, Christina Hulbe, Christian Ohneiser, Craig Stevens, Blair Thomson, Ramunas Stepanauskas, José M. González, Ramiro Logares, Gerhard J. Herndl, Sergio E. Morales*, Federico Baltar***

Correspondence to:

Dr. Federico Baltar,
Email: federico.baltar@univie.ac.at

Dr. Sergio E. Morales,
Email: sergio.morales@otago.ac.nz

This document includes

- **Supplementary Note 1**: Estimations of carbon fixation under the RIS, from the measured ammonium concentrations in the ice basal boundary layer (IBL).
- **Supplementary Note 2**: Shifts in community composition and activity with depth.
- **Supplementary Methods**: Single cell genomics
- **Supplementary Figures** 1 to 17
- **Supplementary References**

Supplementary Notes

Supplementary Note 1. Estimations of carbon fixation under the RIS, from the measured ammonium concentrations in the ice basal boundary layer (IBL).

The rate of ammonium oxidation at each depth was calculated based on the measured ammonium concentrations, and the specific nitrification rate (λ_{nitrif}, the daily rate of ammonium oxidation divided by the corresponding ammonium concentration). As a representative value, we used the world median λ_{nitrif}, 0.195 d^{-1} (*1*). The calculated ammonium oxidation rates are listed in the table below (column 4).

Water column zones	Depth (m)	NH_4^+ (µM)	NH_4^+ oxidation (µM NH_4^+ d^{-1})	Substrate use efficiency (C fixation/NH_4^+ oxidation)	AOA-fueled C fixation (µM C m^{-3} d^{-1})
IBL	30	0.44	0.09	0.09	8.1
V-IL	180	0.05	0.01	0.09	0.9
S-IL	330	0.04	0.01	0.09	0.9

The resulting ammonium oxidation values in the IBL (90 nM NH_4^+ d^{-1}) are in accordance to rates measured in Southern Ocean waters with comparable ammonium concentrations (e.g., AASW with mean 0.7 µM NH_4^+ support a mean ammonium oxidation of 62 nM NH_4^+ d^{-1} (*2*). Unfortunately, no carbon fixation rates were provided in the cited study.

The conversion to carbon fixation was then calculated based on experimentally defined substrate use efficiency. At a value of 0.09 mol C fixed per mol NH_4^+ (*3*), the community in the IBL has the capacity to fix up to 8.1 µmol C m^{-3} d^{-1}. This is a value very close to that measured by Horrigan and colleagues (*4*) beneath the J9 borehole (8.3 µmol C m^{-3} d^{-1}) and is a value in accordance to the total carbon demand under the Ross Ice Shelf (6-12 µmol C m^{-3} d^{-1}), estimated in this study from heterotrophic production rates (**Table 1**).

The above calculations have not considered the second step of nitrification (i.e., nitrite oxidation), and thus a fraction of ammonium-fuelled autotrophy is not accounted for. It should also be noted that these calculations are based on carbon use efficiencies of optimally growing ammonia oxidizing archaeal cultures, and thus should be interpreted as an upper end for autotrophy fuelled by ammonium oxidizing archaea. Even if a rough consideration, they suggest that the measured ammonium concentrations can realistically support a significant fraction of the total dark carbon fixation in the oceanic cavity under the Ross Ice Shelf. As a comparison to existing environmental measurements, the ammonium concentration measured at 200m beneath borehole J9, (the depth from where the C-fixation rates were detected (*4*) was of ~0.7 µM (slightly higher, but in the range of what this study recorded: 0.4 µM, **Table 1**).

Supplementary Note 2. Shifts in community composition and activity with depth

Indicator species analysis on the amplicon data revealed that several species belonging to the phyla differentially enriched under the Ross Ice Shelf, including phyla Chloroflexota, Myxococcota, and Planctomycetota were also signature species of the mid water column (180 m and 330 m), while members of the phyla Marinisomatota and the genus *Nitrosopumilus* were identified as an indicator species of the basal layer beneath the ice (Indval, $p = 0.019 – 0.043$, test statistic > 0.5; **Supplementary Data 1)**.

A similar trend was observed in the relative abundance and transcriptional activity of reconstructed genomes from dominant taxa **(Supplementary Figures 4 & 5)**. In particular, the relative abundance of the genus *Nitrosopumilus* was higher at 30 m, correlating with the highest ammonium concentrations measured in the water column. On the other hand, the relative abundance of heterotrophic taxa (e.g., Planctomycetota, Dehalococcoidia, Verrucomicrobiota) was higher in the deeper samples. Consistent with the increased relative abundance of these phyla at deeper depths, the relative abundance and transcription of CAZyme-encoding genes were also higher in deeper waters **(Figure 4)**. These observations suggest that the most active and abundant community members in the cavity beneath the Ross Ice Shelf shift from from nitrifying autotrophic taxa at 30 m depth to organoheterotrophic bacteria at 180 and 330 m depth. We propose that our observations reflect the influence of increased ammonium concentrations beneath the ice basal layer. However, the resolution of the depth sampling is not sufficient to confirm this transition. This reflects we prioritized a sampling strategy that would ensure

minimum disturbance and contamination, at the cost of being time-intensive and preventing additional sampling depths. Further work is encouraged to further explore the presence and persistence of ammonium concentration gradients inside ice shelf cavities, and their correlation with the composition and activity of below shelf microbial communities.

Supplementary Methods

Single cell genomics. Triplicate seawater samples (1 mL each) were transferred to sterile cryovials containing 100 µL of glyTE (20 mL of 100 × TE buffer pH 8.0, 60 mL Milli-Q water and 100 mL of molecular-grade glycerol) and stored at -80 °C. They were shipped on dry ice for analyses at the Bigelow Laboratory for Ocean Sciences' Single Cell Genomic Center (SCGC).

After thawing, seawater samples were incubated with the SYTO-9 DNA stain (5 µM; Thermo Fisher Scientific) for 10-60 min. Fluorescence-activated cell sorting (FACS) was performed using a BD InFlux Mariner flow cytometer equipped with a 488 nm laser for excitation and a 70 µm nozzle orifice (Becton Dickinson, formerly Cytopeia). The cytometer was triggered on side scatter, and the "single-1 drop" mode was used for maximal sort purity. Sort gate was defined based on particle green fluorescence (proxy to nucleic acid content), light side scatter (proxy to size), and the ratio of green versus red fluorescence (for improved discrimination of cells from detrital particles). Cells were deposited into 384-well microplates containing 600 nL per well of 1x TE buffer and stored at −80ºC until further processing. Of the 384 wells, 317 wells were dedicated for single cells, 64 wells were used as negative controls (no droplet deposition), and 3 wells received 10 cells each to serve as positive controls. The accuracy of droplet deposition into microplate wells was confirmed several times during each sort day, by sorting 3.46 µm diameter SPHERO Rainbow Fluorescent Particles (Sperotech Inc.) and microscopically examining their presence at the bottom of each well. In these examinations, <2% wells did not contain beads and <0.4% wells contained more than one bead. Index sort data was collected using the BD FACS Sortware software. The following laboratory cultures were used in the development of a cell diameter equivalent calibration curve: *Prochlorococcus marinus* CCMP 2389, *Microbacterium* sp., *Pelagibacter ubique* HTCC1062, and *Synechococcus* CCMP 2515. Average cell diameters of these cultures were determined using Mulitisizer 4e (Beckman Coulter). Average light forward scatter of each of the four cultures was determined using the same BD InFlux Mariner settings as in environmental sample sorting and was repeated each day of single cell sorting. We observed a strong correlation between cell diameters and light forward scatter (FSC) among these cultures (*5*). Taking advantage of this correlation, the diameter equivalent of the sorted environmental cells (D) was estimated from a log-linear regression model:

D = 10^(*a* * log10(FSC) - *b*),

where *a* and *b* are empirically derived regression coefficients (*5*).

Prior to genomic DNA amplification, cells were lysed and their DNA was denatured by five freeze-thaw cycles, the addition of 700 nL of a lysis buffer consisting of 0.4 M KOH, 10 mM EDTA and 100 mM dithiothreitol, and a subsequent 10-minute incubation at 20ºC. The lysis was terminated by the addition of 700 nL of 1 M Tris-HCl, pH 4. Single cell whole genome amplification was performed using WGA-X (*5*). Briefly, the 10 µL WGA-X reactions contained 0.2 U µL-1 Equiphi29 polymerase (Thermo Fisher Scientific), 1x Equiphi29 reaction buffer (Thermo Fisher Scientific), 0.4 µM each dNTP (New England BioLabs), 10 µM dithiothreitol (Thermo Fisher Scientific), 40 µM random heptamers with two 3'-terminal phosphorothioated nucleotide bonds (Integrated DNA Technologies), and 1 µM SYTO-9 (Thermo Fisher Scientific) (all final concentrations). These reactions were performed at 45ºC for 12-16 h, then inactivated by a 15 min incubation at 75°C. In order to prevent WGA-X reactions from contamination with non-target DNA, all cell lysis and DNA amplification reagents were treated with UV in a Stratalinker (Stratagene)(*6*). An empirical optimization of the UV exposure was performed in order to determine the length of UV exposure that is necessary to cross-link all detectable contaminants without inactivating the reaction. Cell sorting, lysis and WGA-X setup were performed in a HEPA-filtered environment conforming to Class 1000 cleanroom specifications. Prior to cell sorting, the instrument, the reagents and the workspace were decontaminated for DNA using UV irradiation and sodium hypochlorite solution (*7*). To further reduce the risk of DNA contamination, and to improve accuracy and throughput, Bravo (Agilent Technologies) and Freedom Evo (Tecan) robotic liquid handlers were used for all liquid handling in 384-well plates.

Libraries for SAG genomic sequencing were created with Nextera XT (Illumina) reagents following manufacturer’s instructions, except for purification steps, which were done with column cleanup kits (QIAGEN), and library size selection, which was done with BluePippin (Sage Science, Beverly, MA), with a target size of 500±50 bp. DNA concentration measurements were performed with Quant-iT™ dsDNA Assay Kits (Thermo Fisher Scientific), following manufacturer’s instructions. Libraries were sequenced with NextSeq 500 (Illumina) in 2x150 bp mode using v.2.5 reagents. The obtained sequence reads were quality-trimmed with

Trimmomatic v0.32 (*8*) using the following settings: -phred33 LEADING:0 TRAILING:5 SLIDINGWINDOW:4:15 MINLEN:36. Reads matching the *H. sapiens* reference assembly GRCh38 and a local database of WGA-X reagent contaminants (≥95% identity of ≥100 bp alignments), as well as low complexity reads (containing <5% of any nucleotide) were removed. The remaining reads were digitally normalized with kmernorm 1.05 (http://sourceforge.net/projects/kmernorm) using settings -k 21 -t 30 -c 3 and then assembled with SPAdes v.3.0.0 (*9*) using the following settings: --careful --sc --phred-offset 33. Each end of the obtained contigs was trimmed by 100 bp, and then only contigs longer than 2,000 bp were retained. Contigs matching the *H. sapiens* reference assembly GRCh38 and a local database of WGA-X reagent contaminants (*5*) (≥95% identity of ≥100 bp alignments) were removed. The quality of the resulting genome assemblies was determined using CheckM v.1.0.7 (*10*) and tetramer frequency analysis (*11*). This workflow was evaluated for assembly errors using three bacterial benchmark cultures with diverse genome complexity and %GC, indicating no non-target and undefined bases in the assemblies and average frequencies of mis-assemblies, indels and mismatches per 100 kbp: 1.5, 3.0 and 5.0 (*5*).

16S rRNA gene regions longer than 500 bp were identified using local alignments provided by BLAST against CREST's (*12*) curated SILVA reference database SILVAMod v128 (*13*), and taxonomic assignments were based on a reimplementation of CREST's last common ancestor algorithm. Functional annotation was first performed using Prokka (*14*) with default Swiss-Prot databases supplied by the software. Prokka was run a second time with a custom protein annotation database built from compiling Swiss-Prot (*15*) entries for Archaea and Bacteria.

Supplementary Figures 1-17

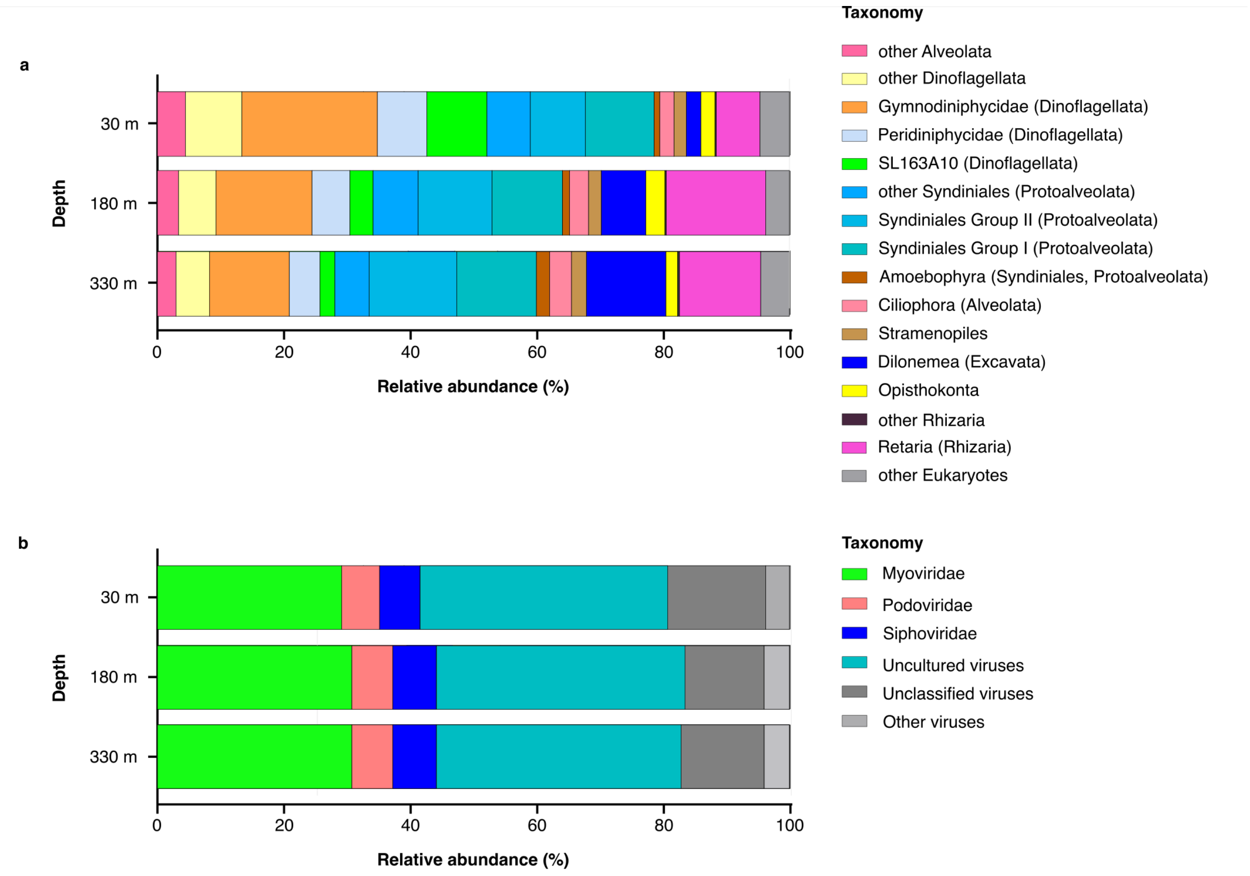

Supplementary Figure 1. Eukaryotic and viral community composition beneath the Ross Ice Shelf, based on metagenomic sequencing. (a) Eukaryotic community composition based on metagenomic 18S rRNA gene reads (miTags) mapped to the SILVA SSU rRNA reference database. **(b)** Viral community composition based on viral signals in assembled metagenomic contigs using VirSorter. For each, averages of replicate sequencing samples are shown. The data used to construct these plots is provided in **Supplementary Data 1** & **2.**

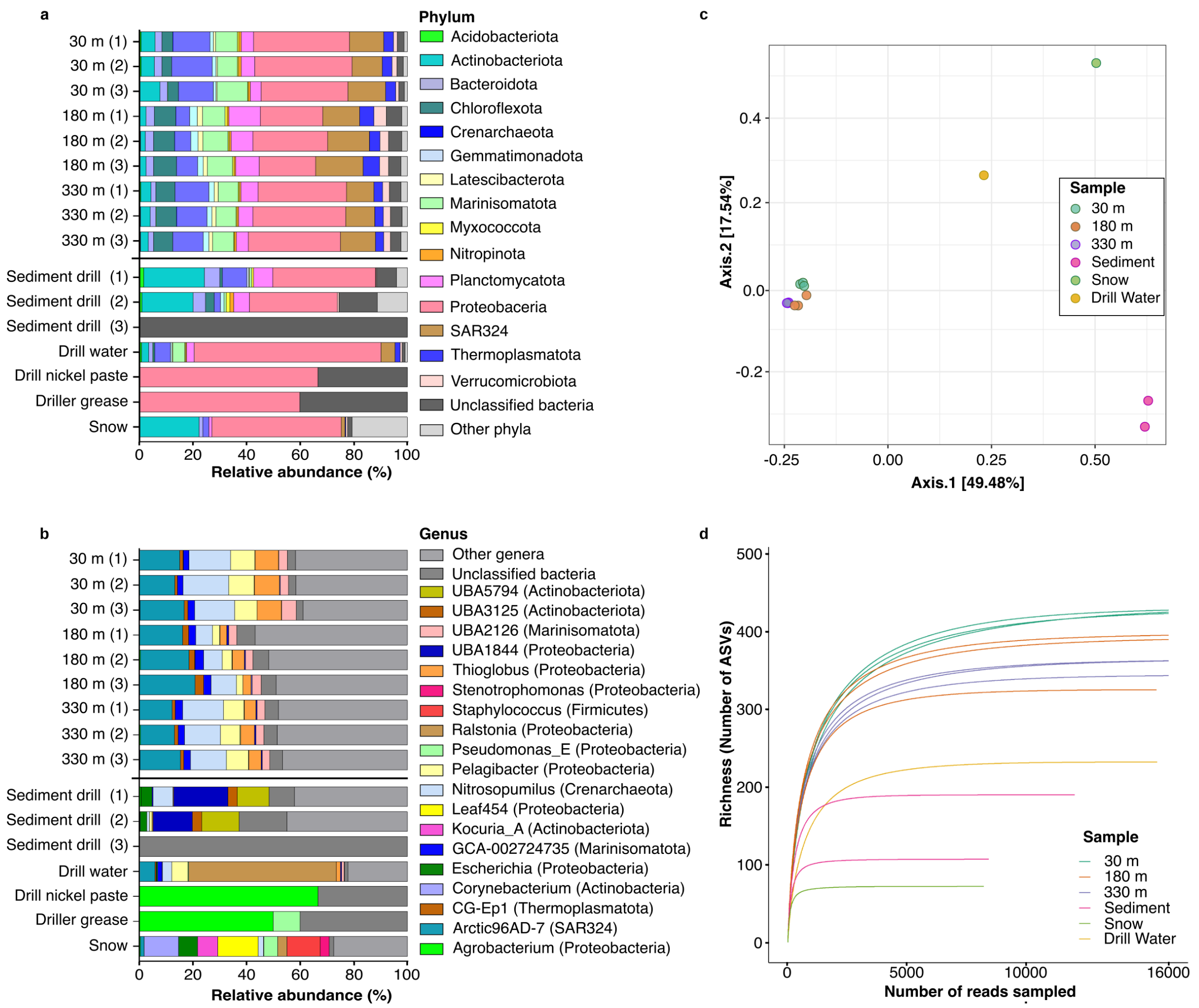

Supplementary Figure 2. 16S rRNA gene amplicon sequencing of the oceanic cavity beneath the Ross Ice Shelf. Community composition of each below-shelf sample and control samples at **(a)** phylum level and **(b)** genus level. The upper nine samples are from the below-shelf water column, whereas the lower seven samples are controls that confirm no significant contamination is present. In all cases, taxonomic classification is based on the Genome Taxonomy Database (GTDB). **(c)** Principal coordinates analysis (PcoA) of community dissimilarity (Bray-Curtis) between the samples. Permanova based on the Bray-Curtis dissimilarities values was used to compare samples and controls and confirm significant differences between groups (**Supplementary Data 3**). **(d)** Rarefaction curves showing number of taxa (ASVs) detected relative to number of sequencing reads. For the three samples not shown in panels **c** and **d** (sediment drill 3, drill nickel paste, driller grease), features count was 20 or less and read count was below 100.

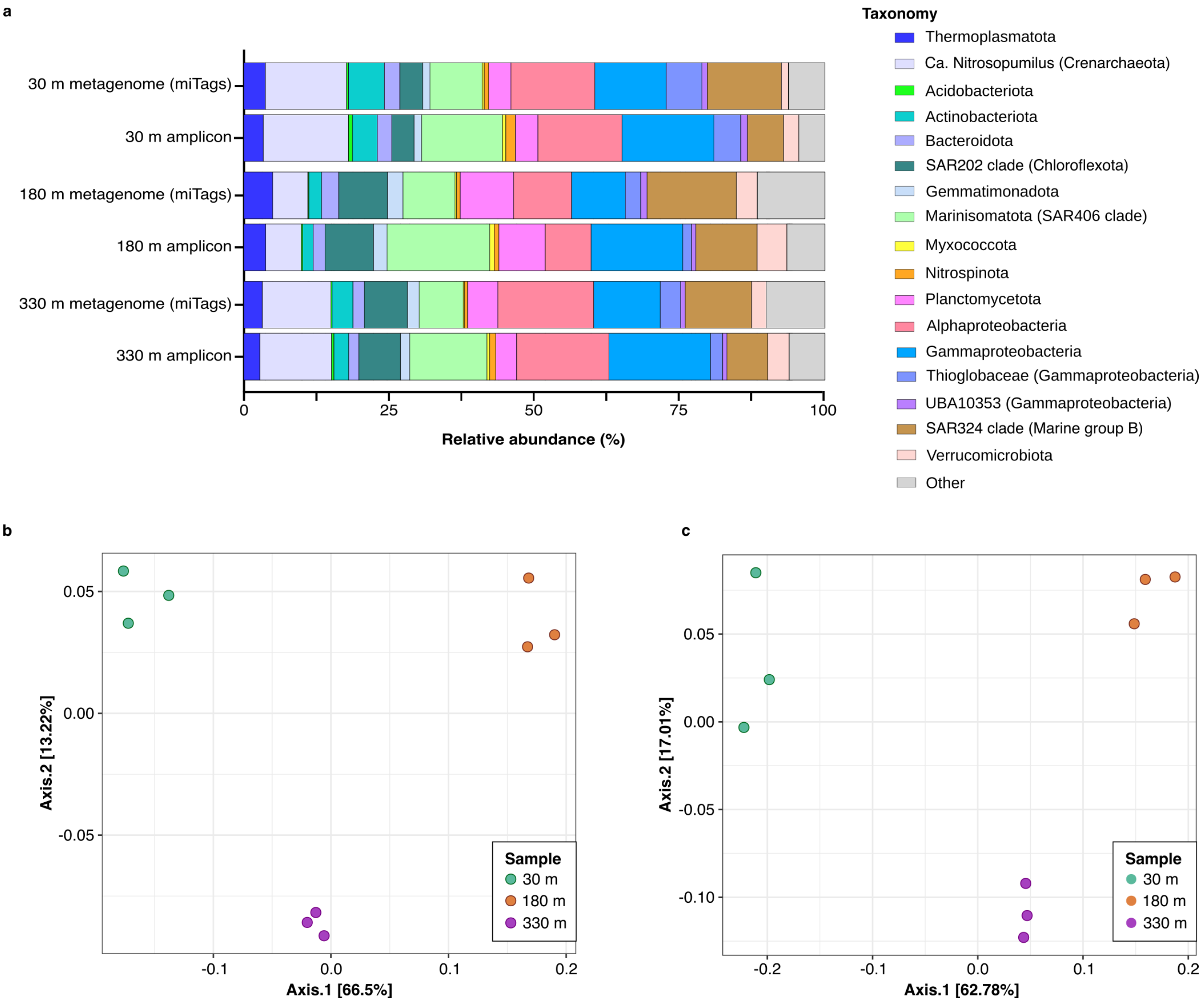

Supplementary Figure 3. Comparison of community composition based on 16S rRNA gene amplicon sequencing and on metagenome 16S rRNA recruited reads. (a) Averaged replicate samples, 16S rRNA gene amplicon sequencing ('amplicon') and metagenome reads from 16S rRNA recruited reads ('miTags'). Genera, families, and classes highlighted throughout the main text were selected for comparison (taxonomic classification is based on the Genome Taxonomy Database, GTDB. **(b-c)** Principal coordinates analysis plot (PCoA) of community dissimilarity (Bray-Curtis) between the three depths sampled, based on **(b)** amplicon sequencing and **(c)** metagenomic miTags (at the species level). Independent Permanova analyses based on the Bray-Curtis dissimilarities values were used to confirm significant differences between depths. As detailed in **Supplementary Data 3**, there are significant differences in richness

between the 30 m and 330 m samples, as well as significant differences in beta diversity between samples.

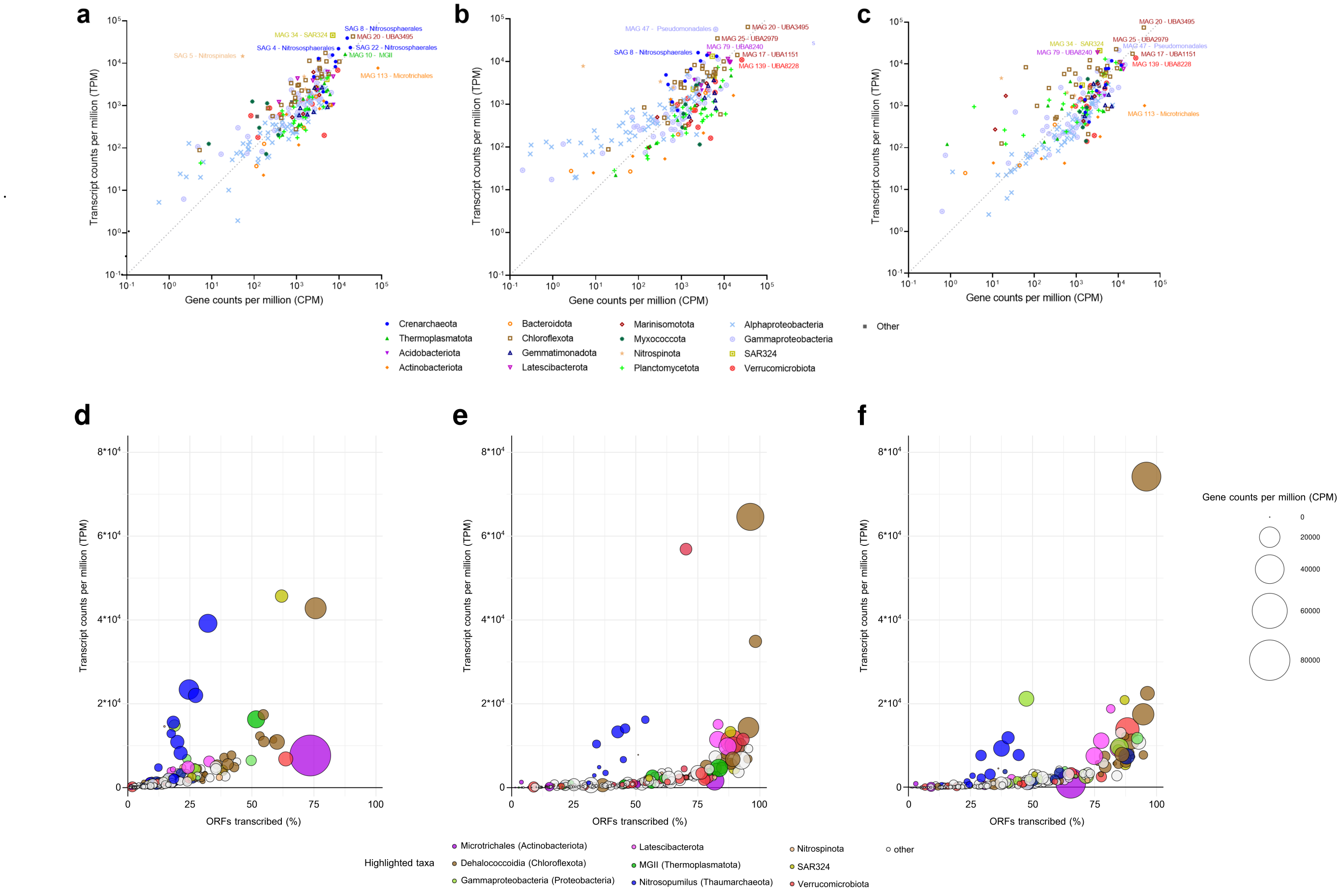

Supplementary Figure 4. Relative abundance of metagenomic and metatranscriptomic short reads mapped to metagenome-assembled genomes and single-amplified genomes. These are expressed as gene counts per million (CPM) and transcripts per million (TPM), respectively. Plots are shown for the reconstructed genomes at **(a, d)** 30 m, **(b, e)** 180 m, and **(c, d)** 330 m deep. In panels **a-b**, the most abundant and/or transcribed genomes are labeled at each depth. In panels **d-f** the size class of each point represents the relative abundance of each genome, while the shading is used to identify the taxa highlighted in the main text. The data used to construct these plots is provided in **Supplementary Data 4**.

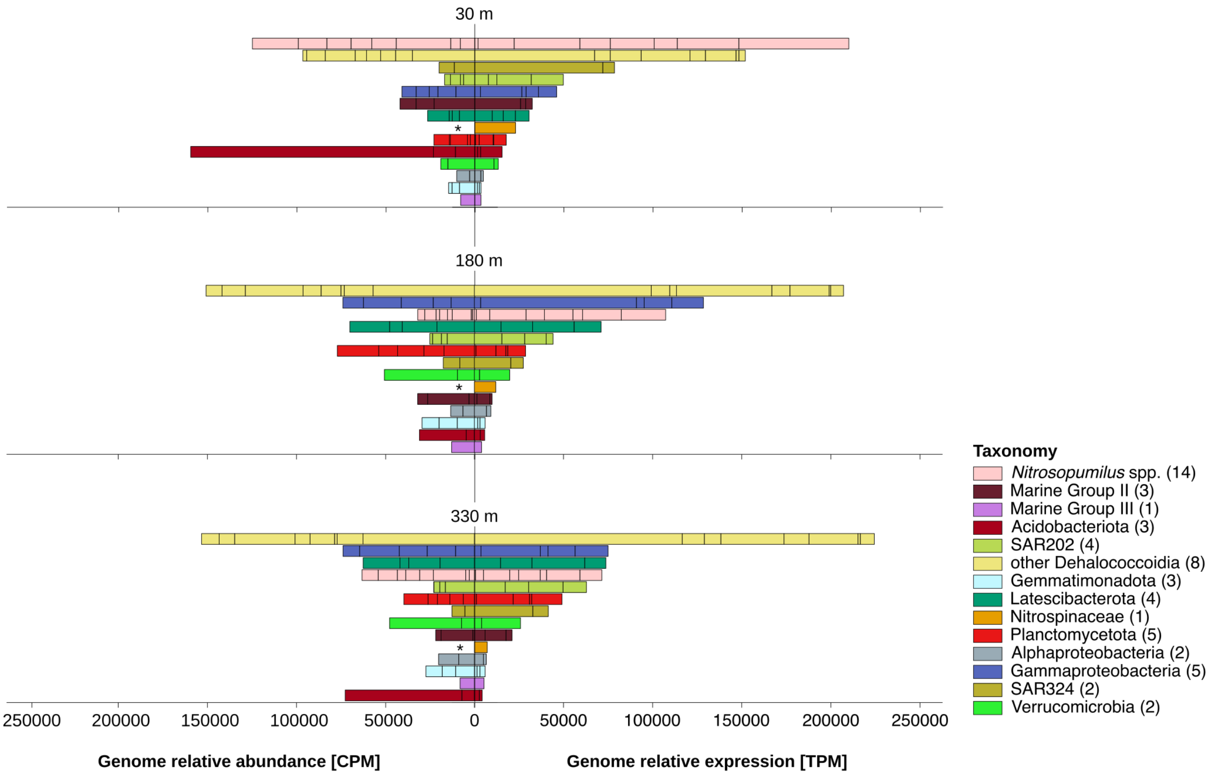

Supplementary Figure 5. Mirror plot of most active and abundant reconstructed genomes, contributing to 50% of total activity and/or abundance in each sample. The number of genomes assigned to each taxonomic category is indicated in brackets. Each genome's coverage and transcription are normalized to total counts (count per million, CPM) and total transcripts (transcripts per million, TPM), respectively. Asterisks mark SAG_5 (order Nitrospinales), whose expression is two orders of magnitude higher than its abundance. The data used to construct these plots is provided in **Supplementary Data 4**.

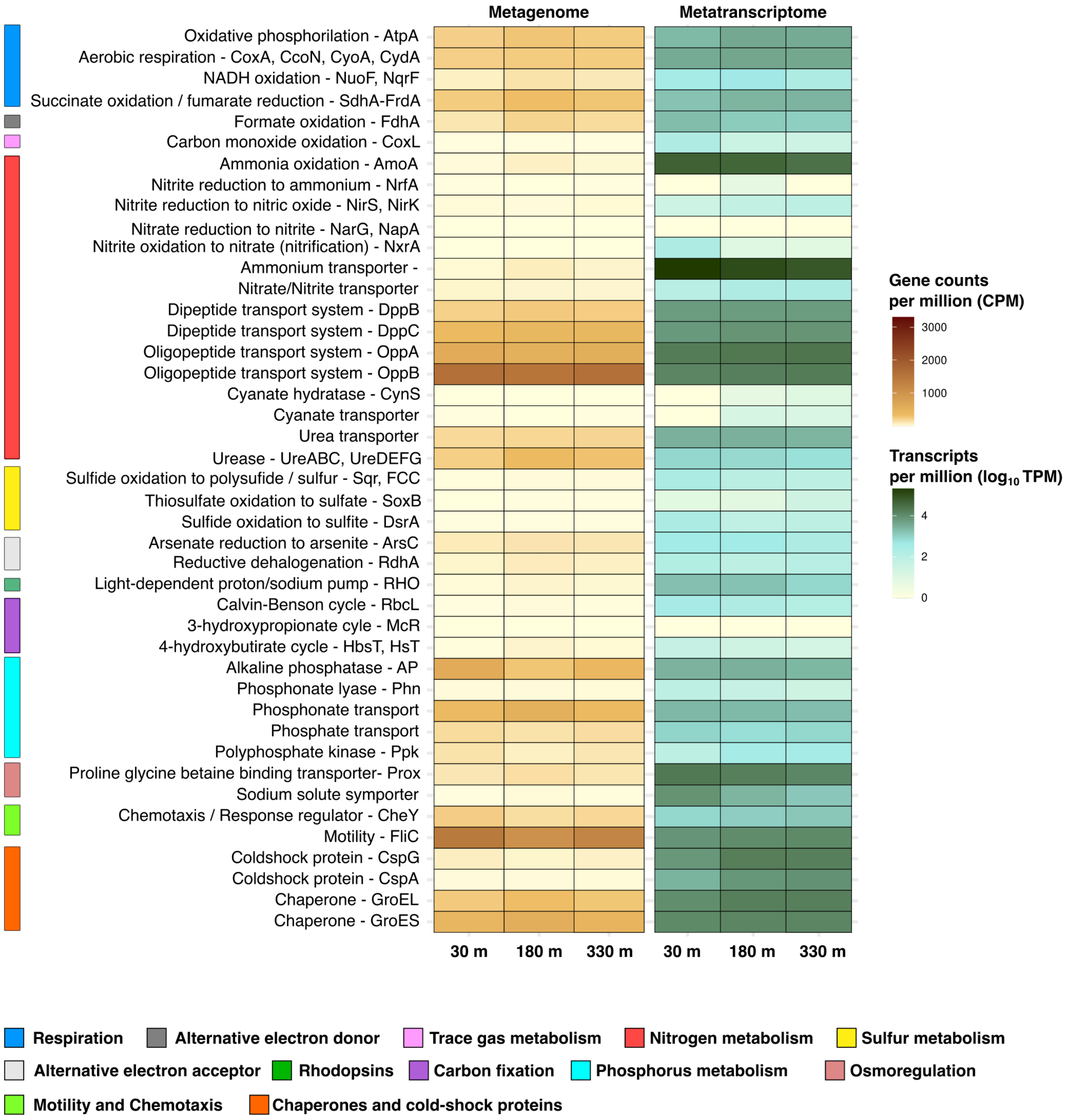

Supplementary Figure 6. Relative abundance of metagenomic and metatranscriptomic short reads mapped to selected genes from metagenomic assembled contigs and single-amplified genomes. Each gene's coverage and transcription is normalized to total counts (count per million, CPM), and total transcripts (transcripts per million, TPM) respectively.

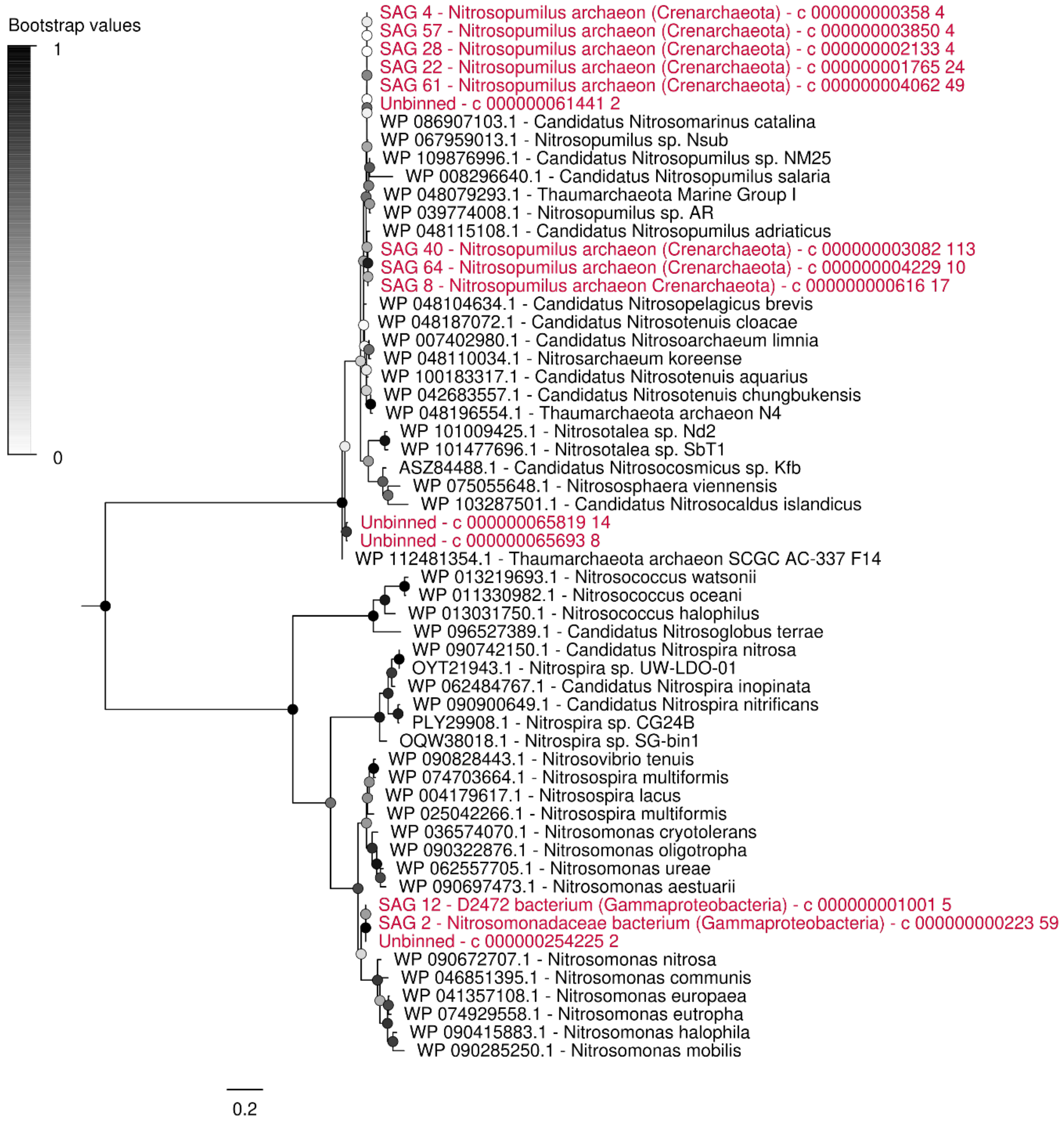

Supplementary Figure 7. Maximum-likelihood phylogenetic tree of ammonia monooxygenase A subunit (AmoA) amino acid sequences. The tree shows sequences from this study's single-amplified genomes and unbinned metagenome contigs alongside representative reference sequences. The tree was constructed using the JTT matrix-based model, used all sites, and was bootstrapped with 50 replicates.

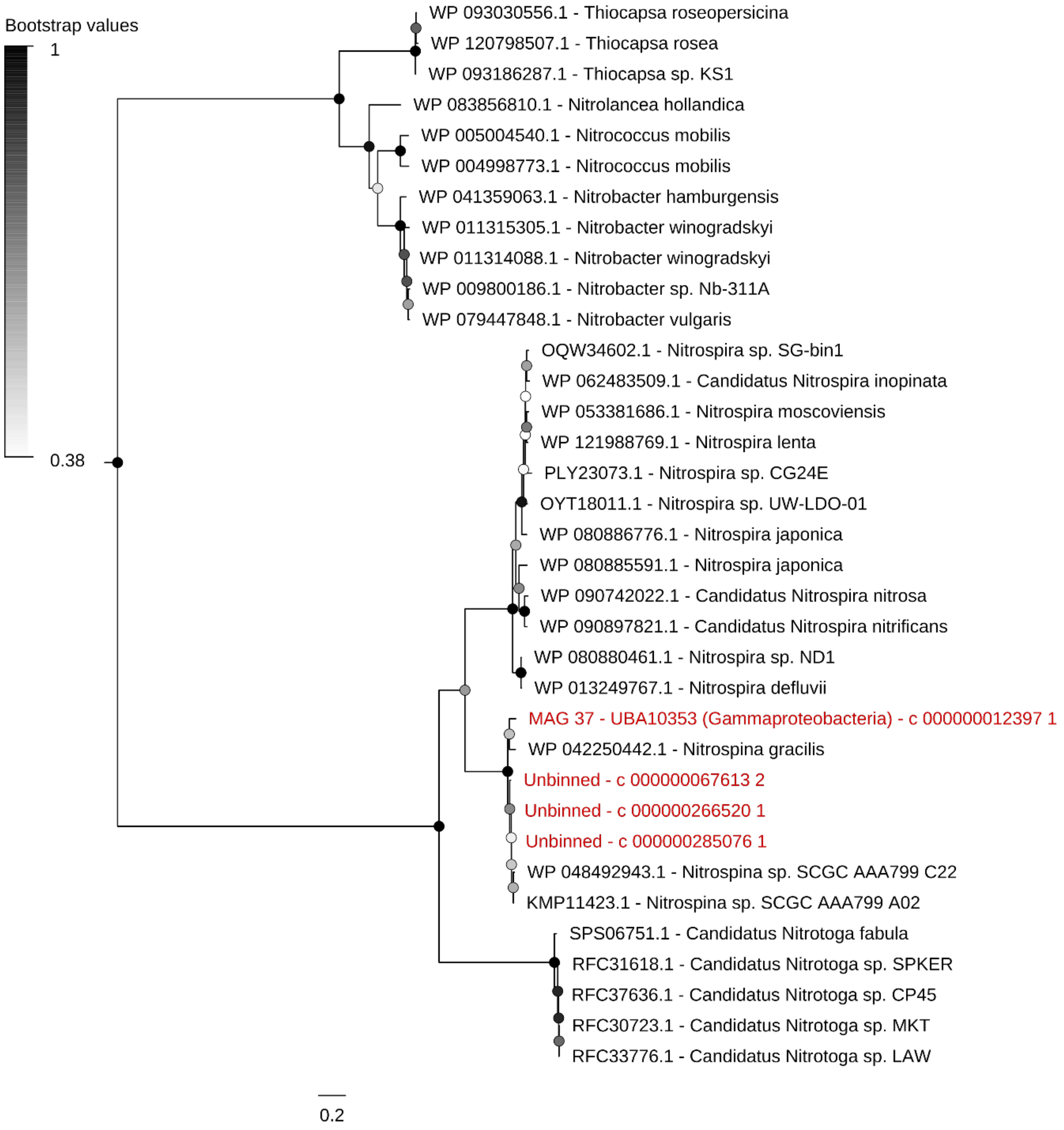

Supplementary Figure 8. Maximum-likelihood phylogenetic tree of nitrite oxidoreductase A subunit (NxrA) amino acid sequences. The tree shows sequences from this study's binned and unbinned metagenome contigs alongside representative reference sequences. The tree was constructed using the JTT matrix-based model, used all sites, and was bootstrapped with 50 replicates.

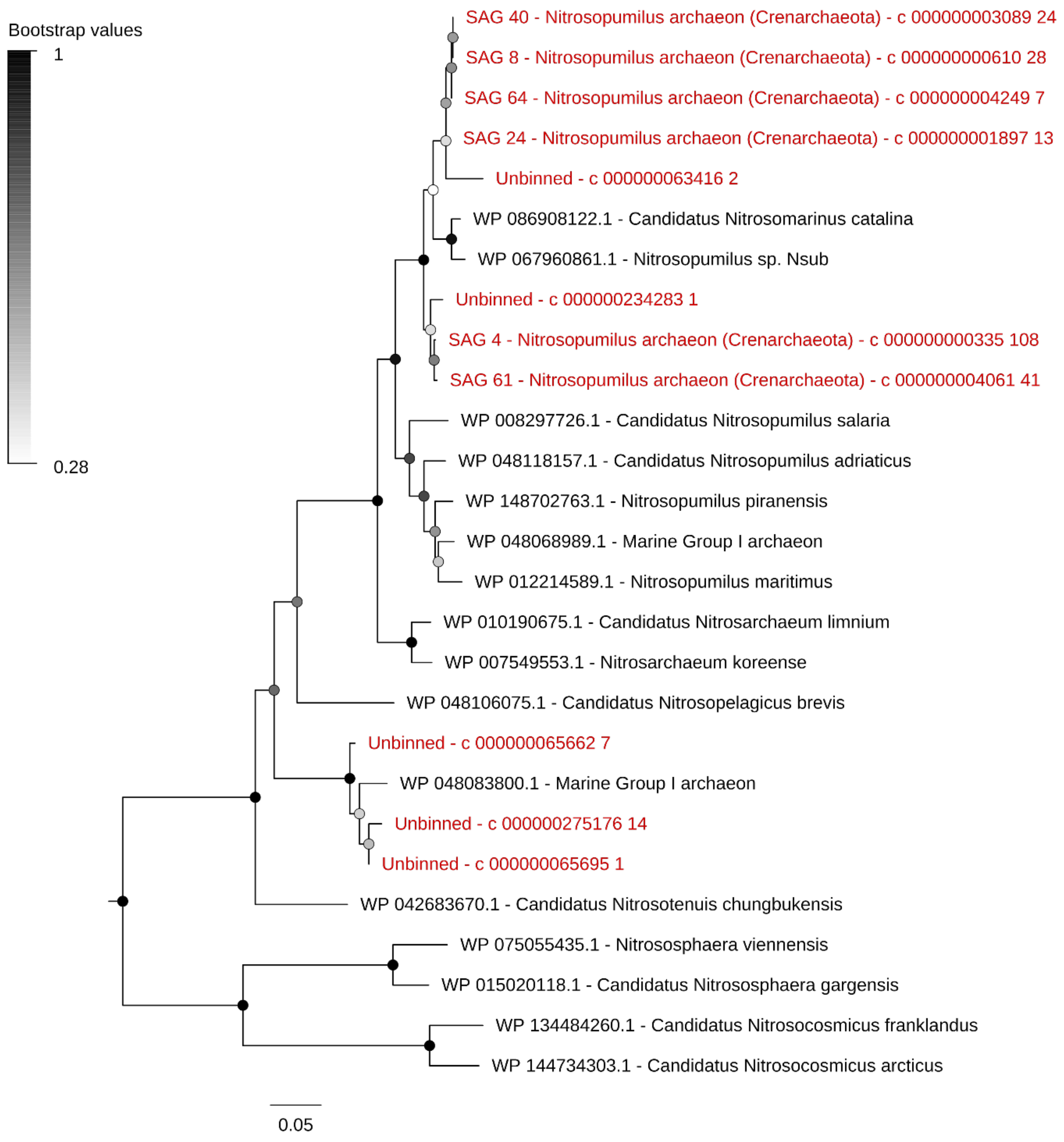

Supplementary Figure 9. Maximum-likelihood phylogenetic tree of thaumarchaeotal 4-hydroxybutyryl-CoA synthetase (HbsT). The tree shows sequences from this study's single-amplified genomes and unbinned metagenome contigs alongside representative reference sequences. The tree was constructed using the JTT matrix-based model, used all sites, and was bootstrapped with 50 replicates.

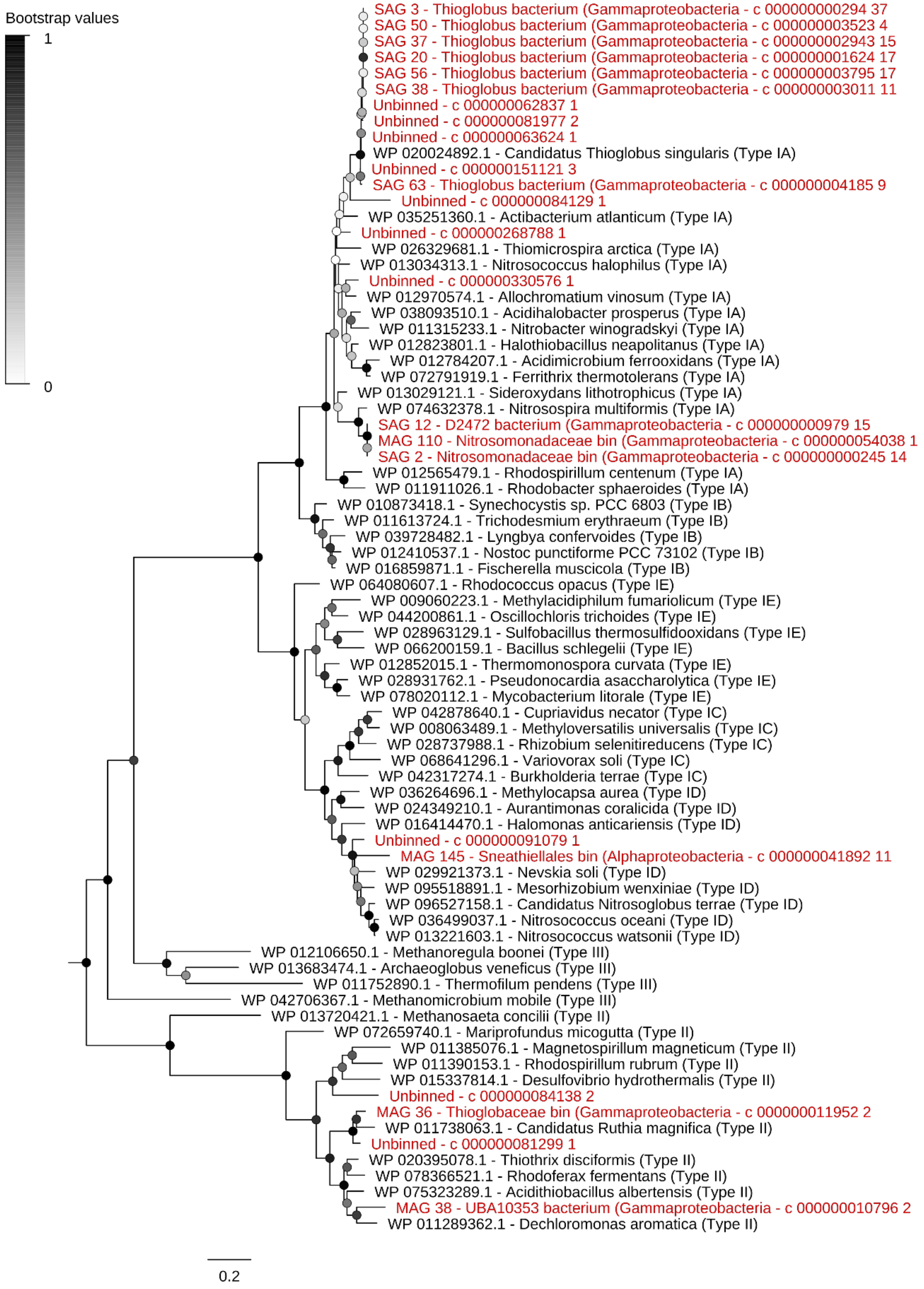

Bootstrap values
1
0
SAG 3 - Thioglobus bacterium (Gammaproteobacteria - c 000000000294 37
SAG 50 - Thioglobus bacterium (Gammaproteobacteria - c 000000003523 4
SAG 37 - Thioglobus bacterium (Gammaproteobacteria - c 000000002943 15
SAG 20 - Thioglobus bacterium (Gammaproteobacteria - c 000000001624 17
SAG 56 - Thioglobus bacterium (Gammaproteobacteria - c 000000003795 17
SAG 38 - Thioglobus bacterium (Gammaproteobacteria - c 000000003011 11
Unbinned - c 000000062837 1
Unbinned - c 000000081977 2
Unbinned - c 000000063624 1
WP 020024892.1 - Candidatus Thioglobus singularis (Type IA)
Unbinned - c 000000151121 3
SAG 63 - Thioglobus bacterium (Gammaproteobacteria - c 000000004185 9
Unbinned - c 000000084129 1
WP 035251360.1 - Actibacterium atlanticum (Type IA)
Unbinned - c 000000268788 1
WP 026329681.1 - Thiomicrospira arctica (Type IA)
WP 013034313.1 - Nitrosococcus halophilus (Type IA)
Unbinned - c 000000330576 1
WP 012970574.1 - Allochromatium vinosum (Type IA)
WP 038093510.1 - Acidihalobacter prosperus (Type IA)
WP 011315233.1 - Nitrobacter winogradskyi (Type IA)
WP 012823801.1 - Halothiobacillus neapolitanus (Type IA)
WP 012784207.1 - Acidimicrobium ferrooxidans (Type IA)
WP 072791919.1 - Ferrithrix thermotolerans (Type IA)
WP 013029121.1 - Sideroxydans lithotrophicus (Type IA)
WP 074632378.1 - Nitrosospira multiformis (Type IA)
SAG 12 - D2472 bacterium (Gammaproteobacteria - c 000000000979 15
MAG 110 - Nitrosomonadaceae bin (Gammaproteobacteria - c 000000054038 1
SAG 2 - Nitrosomonadaceae bin (Gammaproteobacteria - c 000000000245 14
WP 012565479.1 - Rhodospirillum centenum (Type IA)
WP 011911026.1 - Rhodobacter sphaeroides (Type IA)
WP 010873418.1 - Synechocystis sp. PCC 6803 (Type IB)
WP 011613724.1 - Trichodesmium erythraeum (Type IB)
WP 039728482.1 - Lyngbya confervoides (Type IB)
WP 012410537.1 - Nostoc punctiforme PCC 73102 (Type IB)
WP 016859871.1 - Fischerella muscicola (Type IB)
WP 064080607.1 - Rhodococcus opacus (Type IE)
WP 009060223.1 - Methylacidiphilum fumariolicum (Type IE)
WP 044200861.1 - Oscillochloris trichoides (Type IE)
WP 028963129.1 - Sulfobacillus thermosulfidooxidans (Type IE)
WP 066200159.1 - Bacillus schlegelii (Type IE)
WP 012852015.1 - Thermomonospora curvata (Type IE)
WP 028931762.1 - Pseudonocardia asaccharolytica (Type IE)
WP 078020112.1 - Mycobacterium litorale (Type IE)
WP 042878640.1 - Cupriavidus necator (Type IC)
WP 008063489.1 - Methyloversatilis universalis (Type IC)
WP 028737988.1 - Rhizobium selenitireducens (Type IC)
WP 068641296.1 - Variovorax soli (Type IC)
WP 042317274.1 - Burkholderia terrae (Type IC)
WP 036264696.1 - Methylocapsa aurea (Type ID)
WP 024349210.1 - Aurantimonas coralicida (Type ID)
WP 016414470.1 - Halomonas anticariensis (Type ID)
Unbinned - c 000000091079 1
MAG 145 - Sneathiellales bin (Alphaproteobacteria - c 000000041892 11
WP 029921373.1 - Nevskia soli (Type ID)
WP 095518891.1 - Mesorhizobium wenxiniae (Type ID)
WP 096527158.1 - Candidatus Nitrosoglobus terrae (Type ID)
WP 036499037.1 - Nitrosococcus oceani (Type ID)
WP 013221603.1 - Nitrosococcus watsonii (Type ID)
WP 012106650.1 - Methanoregula boonei (Type III)
WP 013683474.1 - Archaeoglobus veneficus (Type III)
WP 011752890.1 - Thermofilum pendens (Type III)
WP 042706367.1 - Methanomicrobium mobile (Type III)
WP 013720421.1 - Methanosaeta concilii (Type II)
WP 072659740.1 - Mariprofundus micogutta (Type II)
WP 011385076.1 - Magnetospirillum magneticum (Type II)
WP 011390153.1 - Rhodospirillum rubrum (Type II)
WP 015337814.1 - Desulfovibrio hydrothermalis (Type II)
Unbinned - c 000000084138 2
MAG 36 - Thioglobaceae bin (Gammaproteobacteria - c 000000011952 2
WP 011738063.1 - Candidatus Ruthia magnifica (Type II)
Unbinned - c 000000081299 1
WP 020395078.1 - Thiothrix disciformis (Type II)
WP 078366521.1 - Rhodoferax fermentans (Type II)
WP 075323289.1 - Acidithiobacillus albertensis (Type II)
MAG 38 - UBA10353 bacterium (Gammaproteobacteria - c 000000010796 2
WP 011289362.1 - Dechloromonas aromatica (Type II)
0.2

Supplementary Figure 10. Maximum-likelihood phylogenetic tree of RuBisCO catalytic subunit (RbcL) amino acid sequences. The tree shows sequences from this study's binned and unbinned metagenome contigs, as well as single-amplified genomes, alongside representative reference sequences. The tree was constructed using the JTT matrix-based model, used all sites, and was bootstrapped with 50 replicates.

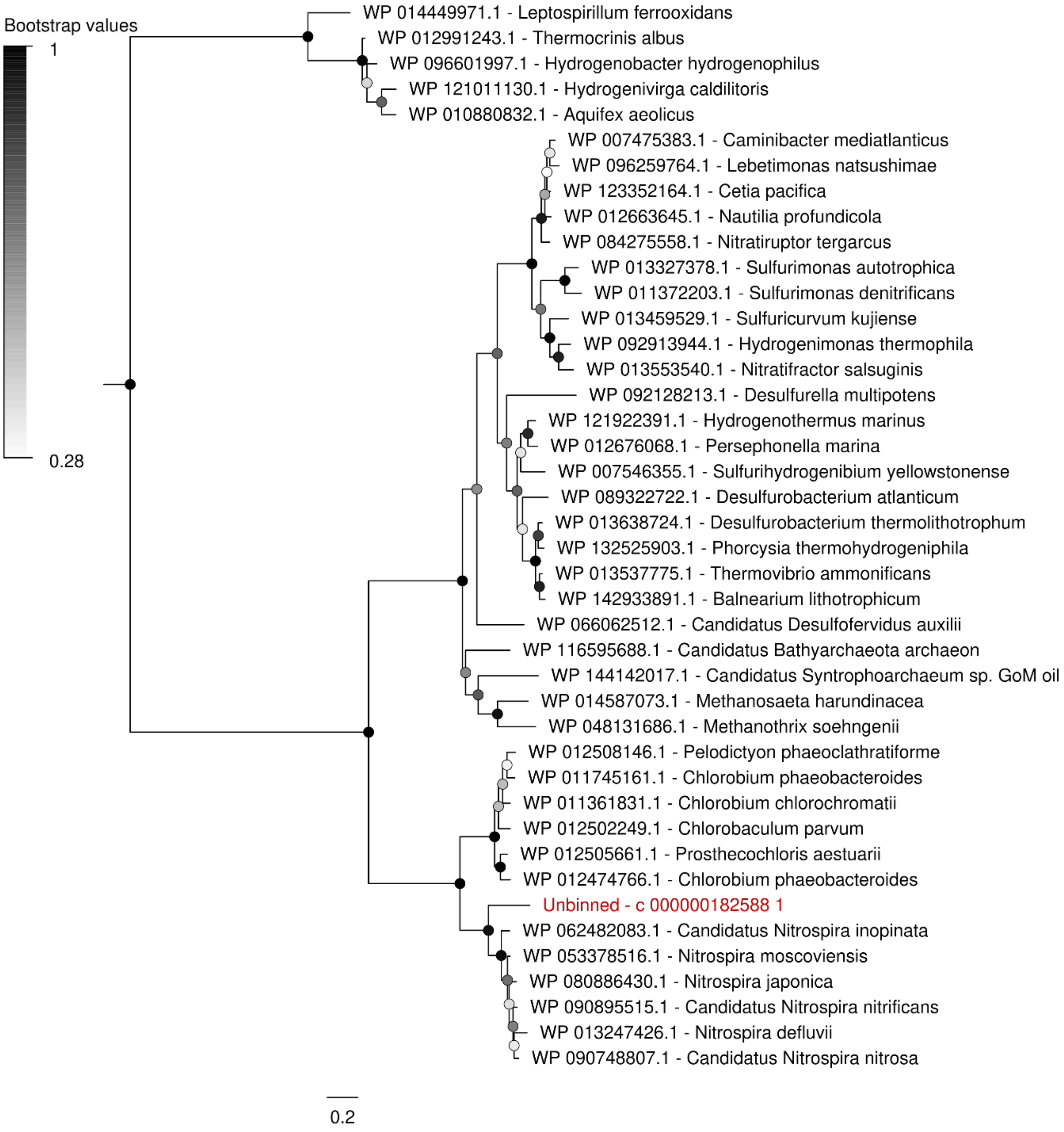

Supplementary Figure 11. Maximum-likelihood phylogenetic tree of ATP-citrate lyase B subunit (AclB) amino acid sequences. The tree shows sequences from this study's unbinned metagenome contigs alongside representative reference sequences. The tree was constructed using the JTT matrix-based model, used all sites, and was bootstrapped with 50 replicates.

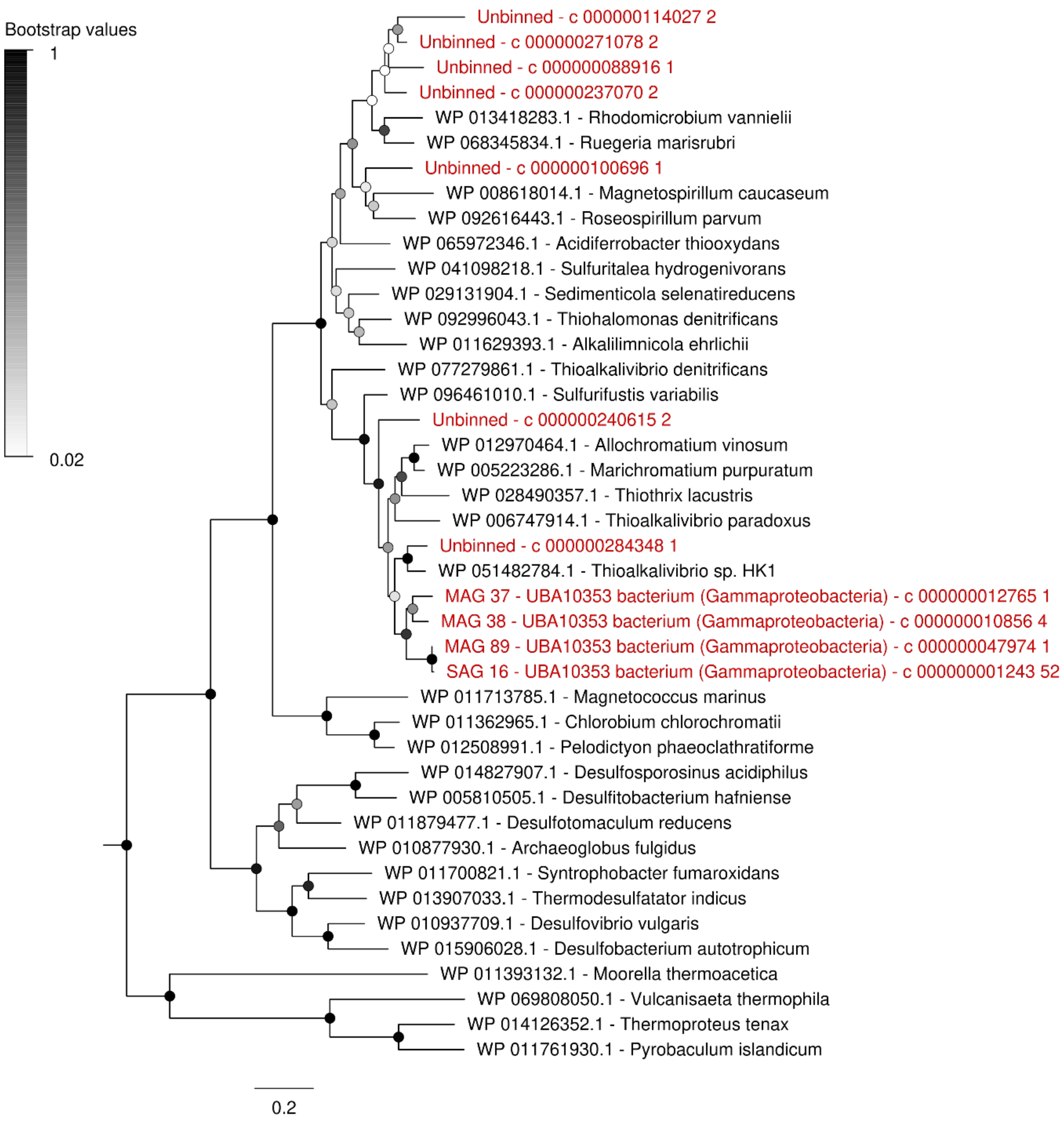

Supplementary Figure 12. Maximum-likelihood phylogenetic tree of dissimilatory sulfite reductase A subunit (DsrA) amino acid sequences. The tree shows sequences from this study's binned and unbinned metagenome contigs, as well as single-amplified genomes, alongside representative reference sequences. The tree was constructed using the JTT matrix-based model, used all sites, and was bootstrapped with 50 replicates. Note all sequences affiliate with known sulfide-oxidising (r-DsrA) clades.

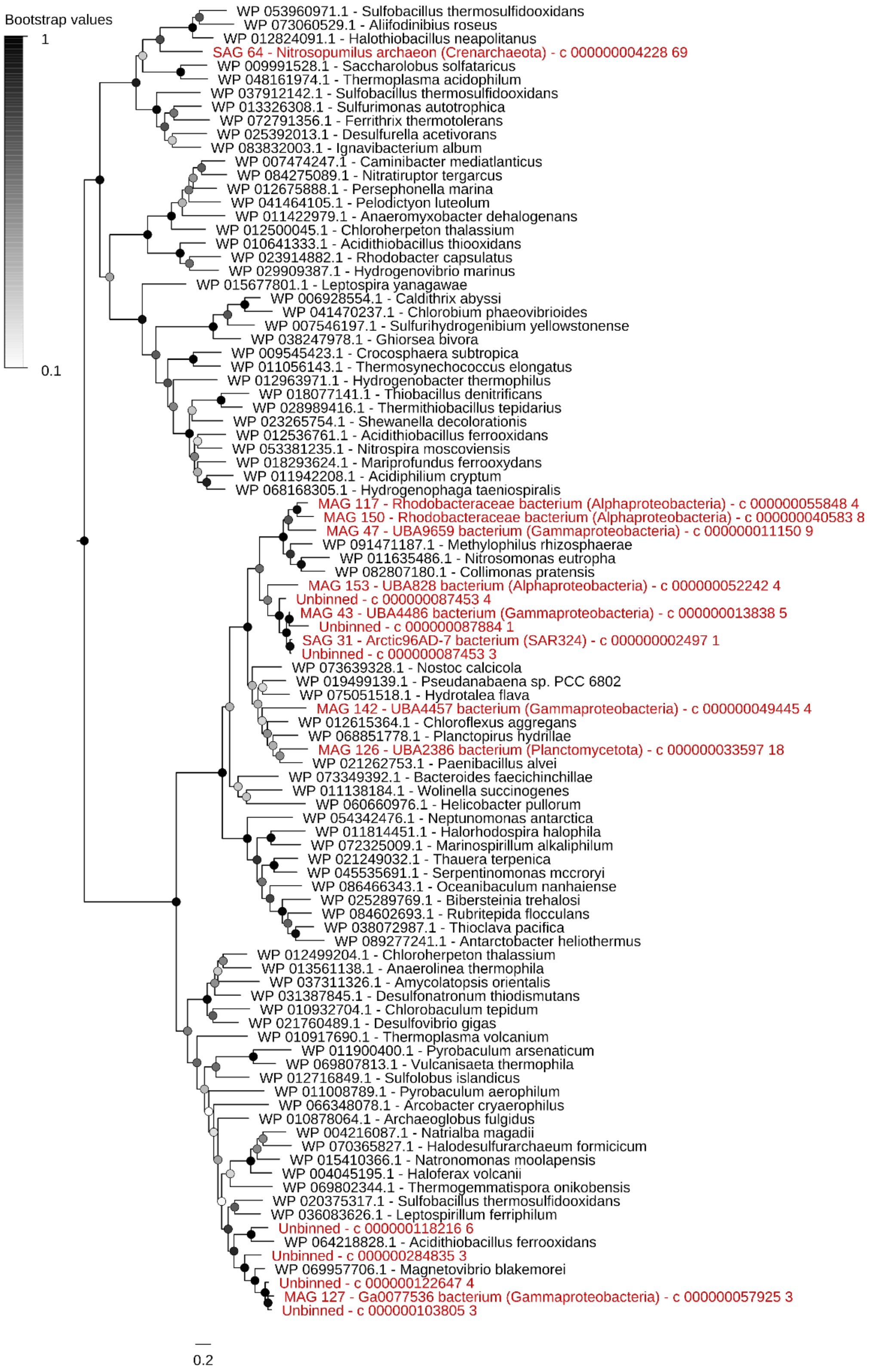

Bootstrap values
1
0.1
WP 053960971.1 - Sulfobacillus thermosulfidooxidans
WP 073060529.1 - Aliifodinibius roseus
WP 012824091.1 - Halothiobacillus neapolitanus
SAG 64 - Nitrosopumilus archaeon (Crenarchaeota) - c 000000004228 69
WP 009991528.1 - Saccharolobus solfataricus
WP 048161974.1 - Thermoplasma acidophilum
WP 037912142.1 - Sulfobacillus thermosulfidooxidans
WP 013326308.1 - Sulfurimonas autotrophica
WP 072791356.1 - Ferrithrix thermotolerans
WP 025392013.1 - Desulfurella acetivorans
WP 083832003.1 - Ignavibacterium album
WP 007474247.1 - Caminibacter mediatlanticus
WP 084275089.1 - Nitratiruptor tergarcus
WP 012675888.1 - Persephonella marina
WP 041464105.1 - Pelodictyon luteolum
WP 011422979.1 - Anaeromyxobacter dehalogenans
WP 012500045.1 - Chloroherpeton thalassium
WP 010641333.1 - Acidithiobacillus thiooxidans
WP 023914882.1 - Rhodobacter capsulatus
WP 029909387.1 - Hydrogenovibrio marinus
WP 015677801.1 - Leptospira yanagawae
WP 006928554.1 - Caldithrix abyssi
WP 041470237.1 - Chlorobium phaeovibrioides
WP 007546197.1 - Sulfurihydrogenibium yellowstonense
WP 038247978.1 - Ghiorsea bivora
WP 009545423.1 - Crocosphaera subtropica
WP 011056143.1 - Thermosynechococcus elongatus
WP 012963971.1 - Hydrogenobacter thermophilus
WP 018077141.1 - Thiobacillus denitrificans
WP 028989416.1 - Thermithiobacillus tepidarius
WP 023265754.1 - Shewanella decolorationis
WP 012536761.1 - Acidithiobacillus ferrooxidans
WP 053381235.1 - Nitrospira moscoviensis
WP 018293624.1 - Mariprofundus ferrooxydans
WP 011942208.1 - Acidiphilium cryptum
WP 068168305.1 - Hydrogenophaga taeniospiralis
MAG 117 - Rhodobacteraceae bacterium (Alphaproteobacteria) - c 000000055848 4
MAG 150 - Rhodobacteraceae bacterium (Alphaproteobacteria) - c 000000040583 8
MAG 47 - UBA9659 bacterium (Gammaproteobacteria) - c 000000011150 9
WP 091471187.1 - Methylophilus rhizosphaerae
WP 011635486.1 - Nitrosomonas eutropha
WP 082807180.1 - Collimonas pratensis
MAG 153 - UBA828 bacterium (Alphaproteobacteria) - c 000000052242 4
Unbinned - c 000000087453 4
MAG 43 - UBA4486 bacterium (Gammaproteobacteria) - c 000000013838 5
Unbinned - c 000000087884 1
SAG 31 - Arctic96AD-7 bacterium (SAR324) - c 000000002497 1
Unbinned - c 000000087453 3
WP 073639328.1 - Nostoc calcicola
WP 019499139.1 - Pseudanabaena sp. PCC 6802
WP 075051518.1 - Hydrotalea flava
MAG 142 - UBA4457 bacterium (Gammaproteobacteria) - c 000000049445 4
WP 012615364.1 - Chloroflexus aggregans
WP 068851778.1 - Planctopirus hydrillae
MAG 126 - UBA2386 bacterium (Planctomycetota) - c 000000033597 18
WP 021262753.1 - Paenibacillus alvei
WP 073349392.1 - Bacteroides faecichinchillae
WP 011138184.1 - Wolinella succinogenes
WP 060660976.1 - Helicobacter pullorum
WP 054342476.1 - Neptunomonas antarctica
WP 011814451.1 - Halorhodospira halophila
WP 072325009.1 - Marinospirillum alkaliphilum
WP 021249032.1 - Thauera terpenica
WP 045535691.1 - Serpentinomonas mccroryi
WP 086466343.1 - Oceanibaculum nanhaiense
WP 025289769.1 - Bibersteinia trehalosi
WP 084602693.1 - Rubritepida flocculans
WP 038072987.1 - Thioclava pacifica
WP 089277241.1 - Antarctobacter heliothermus
WP 012499204.1 - Chloroherpeton thalassium
WP 013561138.1 - Anaerolinea thermophila
WP 037311326.1 - Amycolatopsis orientalis
WP 031387845.1 - Desulfonatronum thiodismutans
WP 010932704.1 - Chlorobaculum tepidum
WP 021760489.1 - Desulfovibrio gigas
WP 010917690.1 - Thermoplasma volcanium
WP 011900400.1 - Pyrobaculum arsenaticum
WP 069807813.1 - Vulcanisaeta thermophila
WP 012716849.1 - Sulfolobus islandicus
WP 011008789.1 - Pyrobaculum aerophilum
WP 066348078.1 - Arcobacter cryaerophilus
WP 010878064.1 - Archaeoglobus fulgidus
WP 004216087.1 - Natrialba magadii
WP 070365827.1 - Halodesulfurarchaeum formicicum
WP 015410366.1 - Natronomonas moolapensis
WP 004045195.1 - Haloferax volcanii
WP 069802344.1 - Thermogemmatispora onikobensis
WP 020375317.1 - Sulfobacillus thermosulfidooxidans
WP 036083626.1 - Leptospirillum ferriphilum
Unbinned - c 000000118216 6
WP 064218828.1 - Acidithiobacillus ferrooxidans
Unbinned - c 000000284835 3
WP 069957706.1 - Magnetovibrio blakemorei
Unbinned - c 000000122647 4
MAG 127 - Ga0077536 bacterium (Gammaproteobacteria) - c 000000057925 3
Unbinned - c 000000103805 3
0.2

Supplementary Figure 13. Maximum-likelihood phylogenetic tree of sulfide:quinone oxidoreductase (Sqr) amino acid sequences. The tree shows sequences from this study's binned and unbinned metagenome contigs, as well as single-amplified genomes, alongside representative reference sequences. The tree was constructed using the JTT matrix-based model, used all sites, and was bootstrapped with 50 replicates.

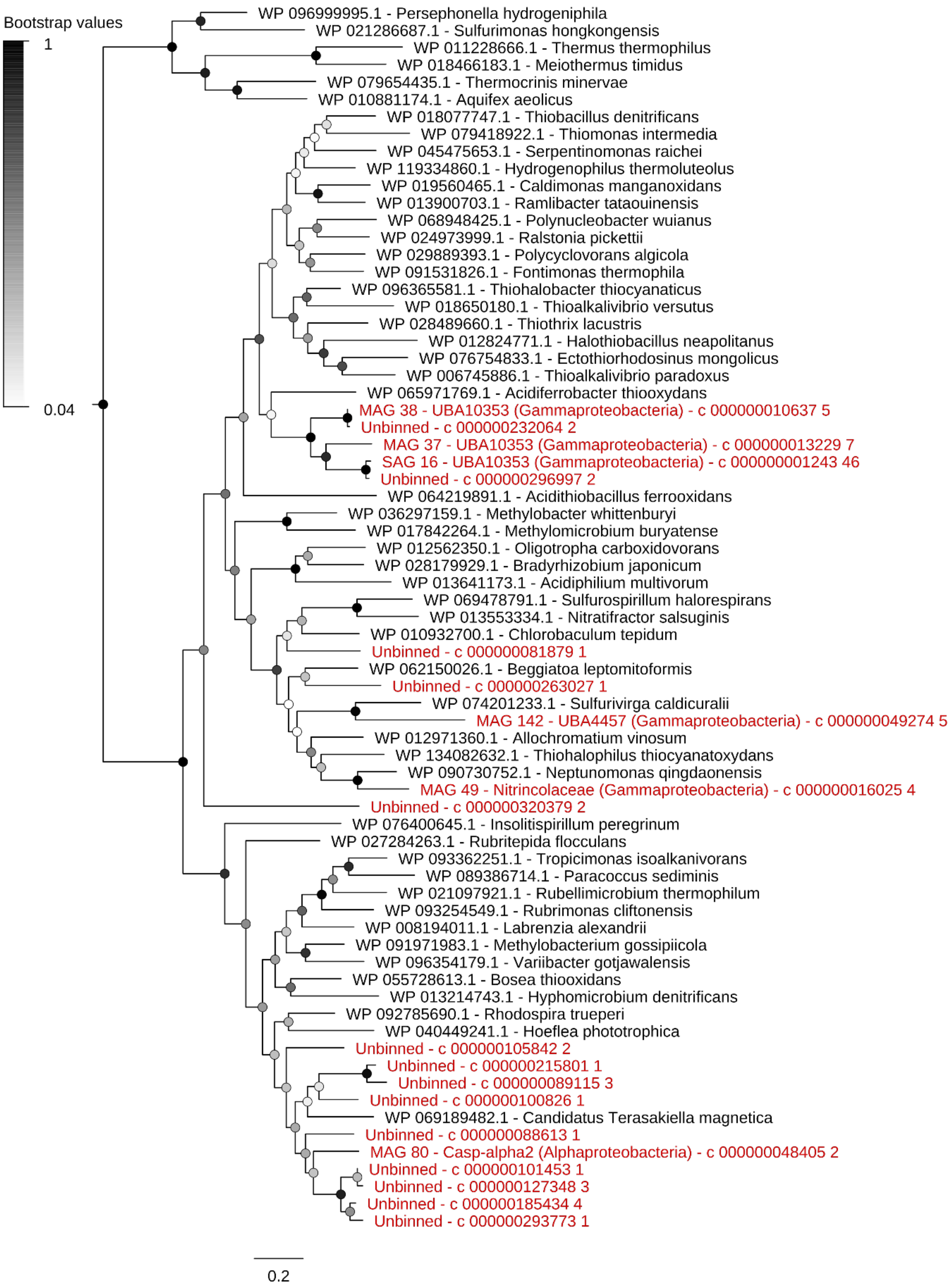

Supplementary Figure 14 Maximum-likelihood phylogenetic tree of thiosulfohydrolase (SoxB) amino acid sequences. The tree shows sequences from this study's binned and unbinned

metagenome contigs, as well as single-amplified genomes, alongside representative reference sequences. The tree was constructed using the JTT matrix-based model, used all sites, and was bootstrapped with 50 replicates.

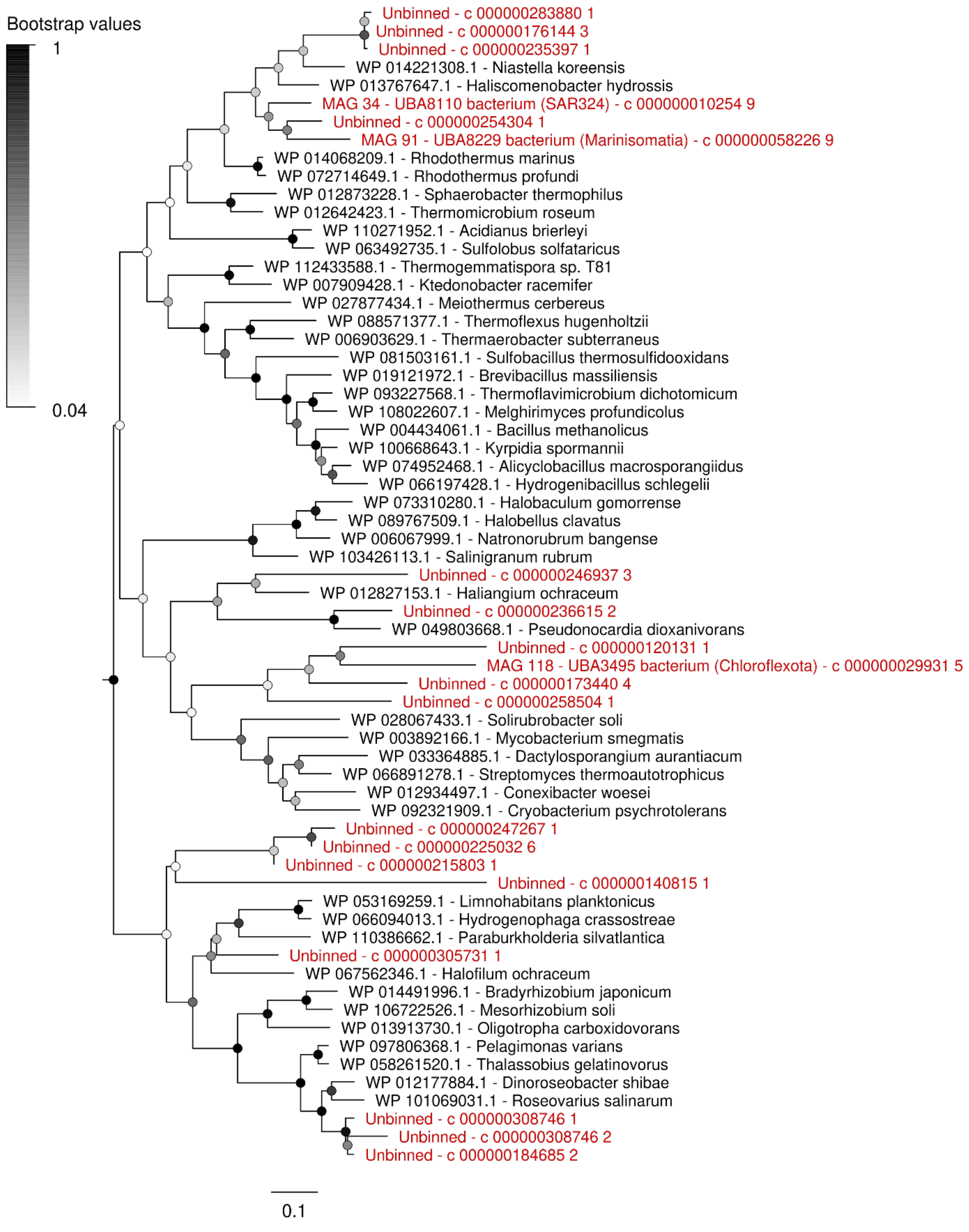

Supplementary Figure 15. Maximum-likelihood phylogenetic tree of carbon monoxide dehydrogenase catalytic subunit (CoxL) amino acid sequences. The tree shows sequences

from this study's binned and unbinned metagenome contigs alongside representative reference sequences. The tree was constructed using the JTT matrix-based model, used all sites, and was bootstrapped with 50 replicates.

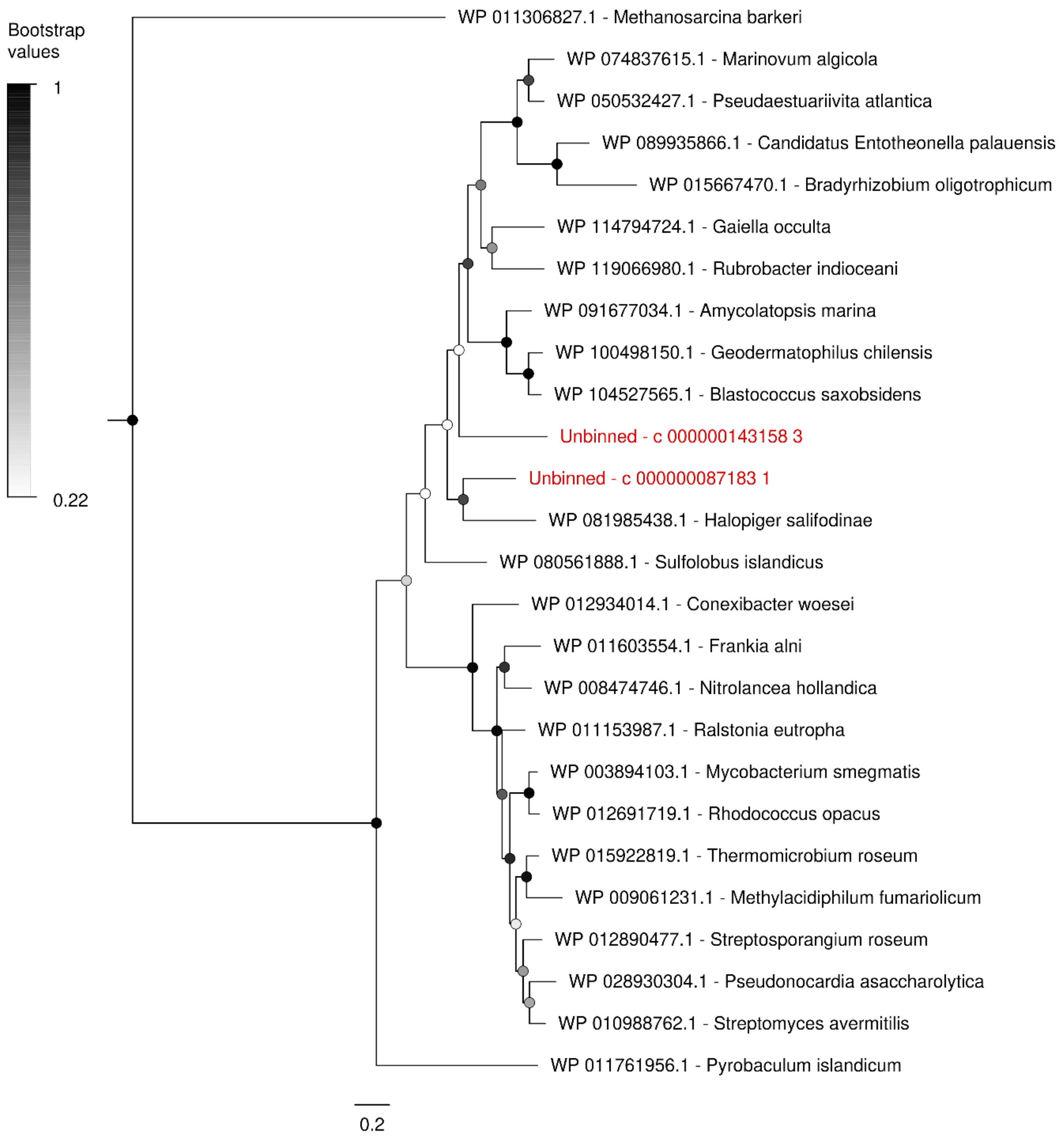

Supplementary Figure 16. Maximum-likelihood phylogenetic tree of group 1h [NiFe]-hydrogenase catalytic subunit (HhyL) amino acid sequences. The tree shows sequences from this study's unbinned metagenome contigs alongside representative reference sequences. The tree was constructed using the JTT matrix-based model, used all sites, and was bootstrapped with 50 replicates. The tree is rooted with group 1f (*Pyrobaculum islandicum*) and group 1k (*Methanosarcina barkeri*) [NiFe]-hydrogenase sequences.

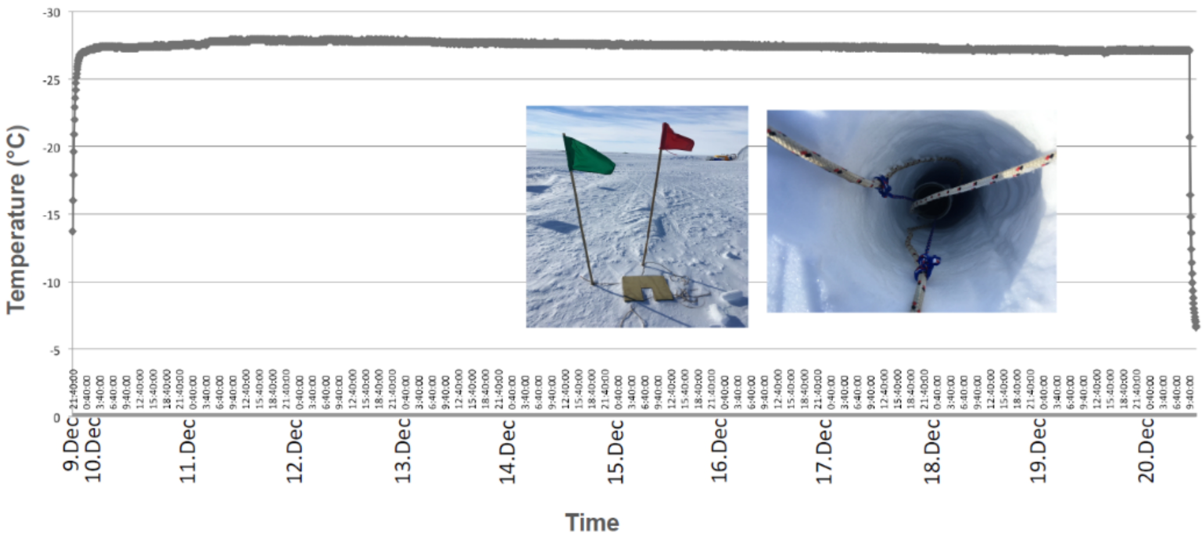

Supplementary Figure 17. Temperature tracking in the 3 m deep borehole where samples were stored. The initial and final changes in temperature are the times when the logger was introduced and retrieved from the borehole. Inserted photos show the borehole where samples were incubated.

Supplementary References

1. A. Yool, A. P. Martin, C. Fernández, D. R. Clark, The significance of nitrification for oceanic new production. *Nat. 2007 4477147*. **447**, 999–1002 (2007).

2. B. B. Tolar, M. J. Ross, N. J. Wallsgrove, Q. Liu, L. I. Aluwihare, B. N. Popp, J. T. Hollibaugh, Contribution of ammonia oxidation to chemoautotrophy in Antarctic coastal waters. *ISME J.* **10**, 2605–2619 (2016).

3. C. Berg, V. Vandieken, B. Thamdrup, K. Jürgens, Significance of archaeal nitrification in hypoxic waters of the Baltic Sea. *ISME J.* **9**, 1319–1332 (2015).

4. S. G. Horrigan, Primary production under the Ross Ice Shelf, Antarctica1. *Limnol. Oceanogr.* **26**, 378–382 (1981).

5. R. Stepanauskas, E. A. Fergusson, J. Brown, N. J. Poulton, B. Tupper, J. M. Labonté, E. D. Becraft, J. M. Brown, M. G. Pachiadaki, T. Povilaitis, B. P. Thompson, C. J. Mascena, W. K. Bellows, A. Lubys, Improved genome recovery and integrated cell-size analyses of individual uncultured microbial cells and viral particles. *Nat. Commun.* **8**, 84 (2017).

6. T. Woyke, A. Sczyrba, J. Lee, C. Rinke, D. Tighe, S. Clingenpeel, R. Malmstrom, R. Stepanauskas, J.-F. Cheng, Decontamination of MDA Reagents for Single Cell Whole Genome Amplification. *PLoS One*. **6**, e26161 (2011).

7. R. Stepanauskas, M. E. Sieracki, *Proc. Natl. Acad. Sci.*, in press.

8. A. M. Bolger, M. Lohse, B. Usadel, Trimmomatic: a flexible trimmer for Illumina sequence data. *Bioinformatics*. **30**, 2114–2120 (2014).

9. S. Nurk, A. Bankevich, D. Antipov, A. A. Gurevich, A. Korobeynikov, A. Lapidus, A. D.

Prjibelski, A. Pyshkin, A. Sirotkin, Y. Sirotkin, R. Stepanauskas, S. R. Clingenpeel, T. Woyke, J. S. Mclean, R. Lasken, G. Tesler, M. A. Alekseyev, P. A. Pevzner, Assembling Single-Cell Genomes and Mini-Metagenomes From Chimeric MDA Products. *J. Comput. Biol.* **20**, 714–737 (2013).

10. D. H. Parks, M. Imelfort, C. T. Skennerton, P. Hugenholtz, G. W. Tyson, CheckM: assessing the quality of microbial genomes recovered from isolates, single cells, and metagenomes. *Genome Res.* **25**, 1043–55 (2015).

11. T. Woyke, G. Xie, A. Copeland, J. M. Gonzalez, C. Han, H. Kiss, J. H. Saw, P. Senin, C. Yang, S. Chatterji, Assembling the marine metagenome, one cell at a time. *PLoS One*. **4**, e5299 (2009).

12. A. Lanzén, S. L. Jørgensen, D. H. Huson, M. Gorfer, S. H. Grindhaug, I. Jonassen, L. Øvreås, T. Urich, CREST – Classification Resources for Environmental Sequence Tags. *PLoS One*. **7**, e49334 (2012).

13. C. Quast, E. Pruesse, P. Yilmaz, J. Gerken, T. Schweer, P. Yarza, J. Peplies, F. O. Glöckner, The SILVA ribosomal RNA gene database project: improved data processing and web-based tools. *Nucleic Acids Res.* **41**, D590–D596 (2012).

14. T. Seemann, Prokka: rapid prokaryotic genome annotation. *Bioinformatics*. **30**, 2068–2069 (2014).

15. The UniProt Consortium, UniProt: the universal protein knowledgebase. *Nucleic Acids Res.* **45**, D158–D169 (2017).
