# Supplementary material for: Phylogenetically and functionally diverse microorganisms reside under the Ross Ice Shelf
Source: Nat Commun. 2022 Jan 10;13:117. doi: 10.1038/s41467-021-27769-5 (PMC8748734; doi:10.1038/s41467-021-27769-5)
Supplement: Supplementary file 3 — Description of Additional Supplementary Files [file 41467_2021_27769_MOESM3_ESM.doc]

**Description of Additional Supplementary Files**

**File Name:** Supplementary Data 1

**Description:** Eukaryotic community composition based on metagenomic 18S rRNA gene reads (miTags) mapped to the SILVA SSU rRNA reference database, expressed as counts per sample.

**File Name:** Supplementary Data 2

**Description:** Viral community composition based on viral signals in assembled metagenomic contigs. Abundance of viral contigs was estimated by recruitment of metagenomic reads to viral contigs and calculation of contig coverage, here expressed as percentage of total viral contig coverage.

**File Name:** Supplementary Data 3

**Description:** Summary of statistical analyses on 16S rRNA gene sequences from amplicon sequencing (ASVs) and metagenomic reads (miTAGs). The following statistical tests are presented:

**Alpha diversity (ASV):** Estimators of the community richness (Chao) and diversity (Shannon index) based on raw and rarefied richness data. One-way anova test and Tukey multiple comparisons of means, with 95% family-wise confidence level.

**Beta diversity (ASV):** Independent permutational analysis of variance (PERMANOVA) based on the Bray-Curtis dissimilarities of below-shelf cavity samples (casts), and of casts and controls. Beta-dispersion test (one-way PERMDISP) using the above Bray-Curtis similarity index.

**Beta diversity (miTags):** Independent permutational analysis of variance (PERMANOVA) based on the Bray-Curtis dissimilarities of samples from the below-shelf cavity and open ocean mesopelagic and bathypelagic polar environments. Beta-dispersion test (one-way PERMDISP) using the above Bray-Curtis similarity index.

**Indval (ASVs):** Indicator Species Analysis between samples from the basal layer (30 m) and mid-column samples (180 m and 330 m). Each IndVal analysis was conducted for each species independently and was not corrected for multiple comparisons.

**Kruskal Wallis (miTags):** non-parametric one-way analysis of variance (Kruskal-Wallis test) based on the Bray-Curtis dissimilarities of samples from the below-shelf cavity and open ocean mesopelagic and bathypelagic environments worldwide.

**MGLM-ANOVA (miTags):** Analysis of Deviance for Multivariate Generalized Linear Model fitting samples from the below-shelf cavity and open ocean mesopelagic and bathypelagic polar environments. Univariate tests were performed on each taxon and corrected for multiple comparisons.

**Indval (miTags):** Indicator Species Analysis between samples from the below-shelf cavity and open ocean mesopelagic and bathypelagic polar environments. Each IndVal analysis was conducted for each species independently and was not corrected for multiple comparisons.

**SIMPER (miTags):** Analysis of similarity percentages based on a Bray-Curtis dissimilarity matrix of samples from the below-shelf cavity and open ocean mesopelagic and bathypelagic polar environments.

**File Name:** Supplementary Data 4

**Description:** Classification, quality, abundance and expression of the metagenome-assembled genomes (MAGs) and single-amplified genomes (SAGs).

**File Name:** Supplementary Data 5

**Description:** Hit counts and fasta sequences for key metabolic genes searched by DIAMOND and HMMs in binned and unbinned assembled reads.

**File Name:** Supplementary Data 6

**Description:** Summary of hit counts and closest affiliations of key metabolic genes searched by DIAMOND in unassembled metagenome and metatranscriptome reads.

**File Name:** Supplementary Data 7

**Description:** Taxonomic affiliation and expression of CAZyme families identified in binned and unbinned assembled reads. GHs, Glycosyl Hydrolases; PL, Polyssacharide lyases; CMBs Carbohydrate-Binding Module; GTs, Glycosyl transferases; CEs, Carbohydrate Esterases.
